# Sonographic Signs of Adenomyosis in Women with Endometriosis Are Associated with Infertility

**DOI:** 10.3390/jcm10112355

**Published:** 2021-05-27

**Authors:** Dean Decter, Nissim Arbib, Hila Markovitz, Daniel S. Seidman, Vered H. Eisenberg

**Affiliations:** 1 Sackler Faculty of Medicine, Tel Aviv University, Tel Aviv 69978, Israel; deanhdecter@gmail.com (D.D.); narbib@gmail.com (N.A.); veredeis@bezeqint.net (H.M.); drmax@bezezqint.net (D.S.S.); 2 Meir Medical Center, Department of Obstetrics and Gynecology, Kfar Saba 4428164, Israel; 3 Sheba Medical Center, Endometriosis Center, Department of Obstetrics and Gynecology, Ramat Gan 5262100, Israel

**Keywords:** adenomyosis, endometriosis, infertility, transvaginal ultrasound, laparoscopic surgery, prevalence

## Abstract

We compared the prevalence of ultrasound signs of adenomyosis in women with endometriosis who underwent surgery to those who were managed conservatively. This was a retrospective study of women evaluated at a tertiary endometriosis referral center who underwent 2D/3D transvaginal ultrasound. Adenomyosis diagnosis was based on the presence of at least three sonographic signs. The study group subsequently underwent laparoscopic surgery while the control group continued conservative management. Statistical analysis compared the two groups for demographics, symptoms, clinical data, and sonographic findings. The study and control groups included 244 and 158 women, respectively. The presence of any, 3+, or 5+ sonographic signs of adenomyosis was significantly more prevalent in the study group (OR = 1.93–2.7, *p* < 0.004, 95% CI; 1.24–4.09). After controlling for age, for all findings but linear striations, the OR for having a specific feature was higher in the study group. Women in the study group with ≥ 5 sonographic signs of adenomyosis had more than twice the risk of experiencing infertility (OR = 2.31, *p* = 0.012, 95% CI; 1.20–4.45). Sonographic signs of adenomyosis are more prevalent in women with symptomatic endometriosis who underwent surgery compared with those who continued conservative management. Women with 5+ findings have a significantly increased risk of infertility. Adenomyosis on ultrasound should be considered in the management decisions regarding these patients.

## 1. Introduction

Adenomyosis is a common disorder defined as the presence of endometrial glands and stroma within the uterine myometrium. It is associated with heavy menstrual bleeding, pain, and infertility [1,2,3]. Known risk factors for adenomyosis include multiparity, early menarche, obesity, and previous uterine surgeries or interventions [4,5,6,7]. The associations between adenomyosis, endometriosis, and infertility are becoming more well-known, but the mechanism by which they might cause infertility is poorly understood [8]. Patients with coexisting, deep infiltrative endometriosis and uterine adenomyosis may constitute a subgroup with particularly poor reproductive outcomes [9,10,11].

Transvaginal ultrasound (TVUS) is now recognized as the first-line imaging modality for women undergoing preoperative evaluation before endometriosis surgery [12,13,14], and it can be similarly adopted for noninvasive diagnosis of adenomyosis [15,16,17]. The most commonly described 2D-TVUS findings for adenomyosis are heterogenous myometrium, abnormal myometrial echo texture, myometrial cysts, a globular and/or asymmetric uterus, ill-defined margins between the endometrium and myometrium (endometrial–myometrial junction (EMJ) or the uterine junctional zone), echogenic linear striations, and focal adenomyomas [18,19,20]. Additionally, 3D-TVUS enables a more detailed assessment of the EMJ zone, which may enhance the diagnosis of adenomyosis [17,21].

We previously evaluated fertility outcomes in infertile women with severe endometriosis and repeated in vitro fertilization (IVF) failures who underwent surgery due to disease exacerbation [22]. Women with severe adenomyosis had lower delivery rates than those without, and women who did not undergo surgery were not studied. The aim of the current study was to determine the prevalence of TVUS signs of adenomyosis in women with symptomatic endometriosis who were referred to our tertiary endometriosis center and subsequently underwent laparoscopic surgery, as compared to those who continued conservative management. Furthermore, we sought to determine the relationship between the presence of adenomyosis (number of sonographic signs) and infertility in these women.

## 2. Materials and Methods

### 2.1. Patients and Setting

This retrospective study included women who underwent 2D/3D-TVUS from May 2011 through September 2015 at our tertiary endometriosis center, and subsequently underwent laparoscopic surgical intervention or continued conservative management.

Women who met the following criteria were included: (1) surgery was performed at our institution; (2) histopathological examination confirmed evidence of endometriosis and/or adenomyosis in women who underwent surgery; (3) women who continued conservative management were matched for age and day of TVUS examination to those who underwent surgery. Women with incomplete TVUS data, who had surgery elsewhere, or who were lost to follow-up were excluded from the analysis.

Demographic information, clinical history, and symptoms were obtained from the electronic hospital records and from outpatient referral documents. These data included age, body mass index (BMI), parity, previous cesarean sections, previous endometriosis surgery, CA125 plasma levels, smoking history (current smoker’s/pack years), fertility treatment and type, and number of previous IVF cycles. Reported symptoms included dysmenorrhea, dyspareunia, urinary and gastrointestinal symptoms, and infertility history.

### 2.2. TVUS Evaluation of Adenomyosis and Endometriosis

All TVUS scans were carried out using a 7.5 MHz probe with 2D/3D capabilities (Voluson GE Medical Systems, Villach, Austria), in a standardized manner by the same imaging expert. 3D volumes were acquired in a standardized way with a single sweep wide angle (120°) in mid-quality setting, with the probe in the midsagittal position. The rendered volume was manipulated to provide a coronal view of the uterus, viewing the endometrial–myometrial junction, myometrium, and the interstitial tubes. Post-processing analysis of stored 2D and 3D images and cineloops was performed at a later date.

**Figure 1:**
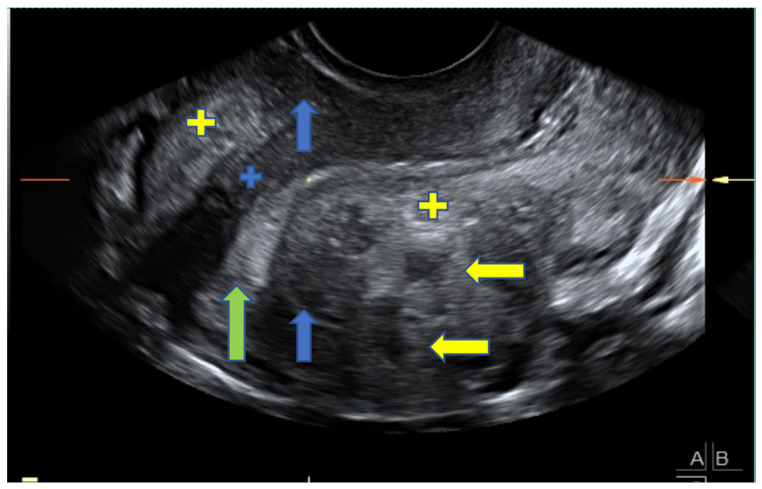
2D transvaginal longitudinal ultrasound image in a symptomatic patient, depicting the uterus with sonographic signs typical of adenomyosis: asymmetrical myometrial thickening (blue arrows), linear striations (blue plus sign), myometrial cysts (yellow arrows), hyperechoic islands (yellow plus sign), and an S-shaped endometrium (green arrow).

A diagnosis of adenomyosis was based on the presence of asymmetrical myometrial thickening (in the absence of fibroids), parallel shadowing, myometrial cysts, hyperechoic islands, irregular EMJ, linear striations, and localized adenomyomas. An adenomyoma was defined as a nodular, heterogeneous myometrial mass with ill-defined borders. These findings were chosen because they are recognized, reliable morphological sonographic markers for adenomyosis [19,20,23,24] and can be differentially diagnosed from fibroids [24]. Figure 1 shows a representative image depicting adenomyosis.

Accuracy was evaluated against the pathology report when available for patients who underwent hysterectomy. However, since most patients did not undergo hysterectomy and in order to increase accuracy, adenomyosis was diagnosed based on a combination of 3 or more findings on TVUS, which was a common procedure at the time [19,20,23,24]. Severe adenomyosis was defined as a combination of 5 or more sonographic findings. Ultrasound diagnosis of endometriosis was based on the presence of ovarian endometriomas, deeply infiltrative endometriotic nodules, signs of pelvic adhesions (kissing ovaries or absent sliding of viscera), or overt tubal disease [12,13,14,17], which was standard practice at the time.

### 2.3. Laparoscopic Surgeries

All laparoscopic surgeries were performed by a multidisciplinary team of surgeons who specialize in laparoscopic surgery for advanced endometriosis. After failure of medical treatment, patients were referred for surgery in the presence of severe intractable symptoms, endometrioma larger than 4 cm, deep infiltrating endometriosis, involvement of other organs (ureter, bladder, rectum, or colon) or following repeated IVF failures. Patients with severe symptoms, findings on pelvic examination and/or US evaluation, and/or infertility, were advised to have surgery before attempting to conceive (spontaneously when relevant or via IVF). Factors such as patient age, previous surgery, and ovarian reserve were included in the decision. Ultimately, the decision to continue medical treatment, to try to achieve pregnancy, or to undergo surgery was based on the patient’s preference, after discussion with the caregiving team.

The severity of endometriosis at surgery was evaluated based on the Revised American Society for Reproductive Medicine (ASRM) Classification [25]. Histopathology reports were reviewed.

### 2.4. Statistical Analysis

Statistical analysis was performed using SPSS software, version 21 (SPSS Inc., IBM corporation, Chicago, IL, U.S.). Continuous variables were expressed as mean ± SD or median depending on whether they were normally distributed or not, while categorical variables were expressed as frequency and percentage. Fisher’s exact test was used to detect differences in percentages and the Student’s *t*-test was used to compare means. Associations between various demographic, symptomatic and clinical variables, disease severity at surgery, and the presence of adenomyosis on ultrasound were assessed using logistic regression. Univariate and multivariate analyses were performed. All analyses were performed when there was at least one ultrasound finding, 3+, and 5+ sonographic findings. The association between sonographic findings of adenomyosis and demographic variables significant in univariate analysis was assessed using logistic regression for 2 models: without adjustment for variables and with adjustment for age. Statistical significance was set at *p* < 0.05.

## 3. Results

### 3.1. Demographic and Clinical Characteristics

The study included 402 women, mean age of 32.7 ± 6.6 years and mean BMI of 23.3 ± 4.9 kg/m^2^; 244 (60.7%) had surgery and 158 (39.3%) underwent conservative management (Table 1). Demographic data, medical history, and symptoms of the women who underwent surgery were compared to those who underwent conservative management (Table 1). Women who underwent surgery were of higher parity, there was a nonsignificant trend to report more symptoms and they were less likely to be infertile, and they had a higher CA125 level. There were no other statistically significant differences.

### 3.2. Ultrasound Findings—Endometriosis and Adenomyosis

The prevalence of sonographic findings suggestive of adenomyosis in women undergoing surgery versus those who did not is presented in Table 2. The presence of any sonographic feature of adenomyosis was more prevalent among women who underwent surgery compared to those who did not. This difference was consistently significant whether we compared based on the presence of at least 1, 3+, or 5+ findings, or mean number of findings. This reached statistical significance for all but linear striations and focal adenomyomas. The prevalence of sonographic findings of adenomyosis was found to increase with age in both groups and for all categories of findings (1, 3+ findings, 5+ findings, or mean number of findings).

The association between sonographic findings of adenomyosis and demographic variables was explored using multivariable analysis and logistic regression without adjustment for variables and with adjustment for age. These results are presented in Table 3. For all findings except linear striations and focal adenomyomas, the odds ratio (OR) for having a specific feature was higher in women undergoing surgery as compared to those who did not. All associations remained significant after adjusting for age, but to a lesser extent. When adjusting for age and parity these associations were not significant.

We did not find a significant association between the number of sonographic findings and the presence or severity of clinical symptoms. In an attempt to determine the severity of adenomyosis based on ultrasound findings, we stratified the number of adenomyosis findings into 5+ or 3+ in the group of women who underwent surgery and performed logistic regression (Table 4). There was no significant association when 3+ findings were considered. However, women with 5+ findings suggestive of adenomyosis had a significantly higher risk of infertility (OR = 2.31, *p* = 0.012, 95% CI; 1.20–4.45).

### 3.3. Surgeries

At surgery, 106 (45.5%) of the 244 women had endometriomas, 12 (4.9%) had full-thickness bladder nodules, 81 (37.3%) had rectovaginal/posterior fornix nodules, 68 (34.3%) had pouch of Douglas obliteration, 50 (24.6%) had recto-sigmoid nodules, 22 (12%) had deep bowel nodules, and 102 (56.7%) had uterosacral ligament involvement.

The mean disease severity (ASRM) score at surgery was 46.1 ± 35.9 (range 1–148). The median ASRM stage was 4 (range 1–4), with 16% at stage I, 4.3% stage II, 20.2% stage III and 59.6% at stage IV disease. Of the 244 women, 33 (13.5%) underwent a hysterectomy. Adenomyosis was confirmed in 27 specimens. These results provide a sensitivity of 92.6%, specificity of 75%, and accuracy of 88.6% for TVUS diagnosis of adenomyosis. Endometriosis was histologically confirmed in all of the women. We did not find an association between sonographic findings of adenomyosis and disease severity based on the ASRM score.

## 4. Discussion

In this study, we found a higher prevalence of sonographic findings of adenomyosis in women undergoing laparoscopic surgery for endometriosis, when compared to women who continued conservative management. This held true when compared on the presence of at least 1 finding, 3+, 5+ (severe adenomyosis), or mean number of sonographic findings. All sonographic findings studied, except linear striations and focal adenomyomas, were more prevalent in the surgery group. In addition, we found that women with 5+ sonographic findings had a significantly higher risk of infertility, thus revealing a strong association between adenomyosis and infertility.

Adenomyosis is frequently reported to be encountered with other concomitant uterine conditions such as fibroids (which was not a common finding in our study), or endometriosis. Dior et al. found that women with sonographic evidence of adenomyosis were more likely to have stage IV or markers of severe endometriosis [26]. They proposed that sonographic signs of adenomyosis may be used in the assessment of and predicting the severity of endometriosis [27]. Capezzuoli et al. found that 21.2% of women with confirmed endometriosis referred for infertility also had adenomyosis [28]. Our study utilized a high resolution TVUS device with an expert sonographer; therefore, we think that the diagnosis was probably of high validity.

Our study did not find an association between adenomyosis and parity, differentiating from previous older studies. Pinzauti et al. evaluated women younger than 30 years without prior history of endometriosis or prior uterine surgeries who were referred for TVUS and reported a 34% incidence of diffuse adenomyosis [29]. Although the pathogenesis of adenomyosis has been debated in the past, it was commonly believed that prior mechanical damage (i.e., prior pregnancy, uterine surgeries) to the endometrial–myometrial junction contributed to its development in older women [4,5,6,7,8]. These recent studies are more in line with our findings, suggesting that parity is not as important as previously thought in the prevalence of adenomyosis. This suggests that parity may not be a risk factor of adenomyosis, particularly when women have concomitant endometriosis. Thus, physicians caring for young women should not necessarily omit adenomyosis from their differential due to age, but should assess for the signs of adenomyosis on TVUS.

A meta-analysis evaluated IVF outcomes in women with adenomyosis and found a 68% decrease in the likelihood of clinical pregnancy with IVF and more than double the risk of miscarriage [30]. In our study, the rate of IVF treatment utilization before surgery was high in both groups. The reason for this may lie in the decision-making process regarding treatment. The women who continued conservative treatment, rather than surgery, subsequently underwent more infertility treatments. Additionally, it is possible that women who underwent surgery had already conceived at least once and thus, the immediate concern for fertility preservation was not as strong and they were more amenable to the surgical option.

The impact of adenomyosis on fertility has been debated in the literature. Studies report an inconsistent range of adenomyosis prevalence, likely due to the lack of use of a consistent diagnostic protocol. Adenomyosis was linked to a 28% reduction in IVF success rate, as reported by Vercellini et al.; however, these results lack the association to sonographic findings [30]. Mavrelos et al. evaluated the impact of adenomyosis sonographic findings to the success rate of IVF and reported that any one finding was associated with a reduced success rate [31]. Furthermore, women with 4+ findings had a 50% reduction in IVF success rate, independent of age and ovarian reserve. These data support our findings that associate infertility to an increasing number (5+) of sonographic findings.

In the past, TVUS was not viewed as a suitable option for diagnosis of adenomyosis, and surgery was considered the gold standard for diagnosis; however, patient management is often based on ultrasound findings alone even though no verified agreement exists [32]. With the advancements of sonographic and MRI techniques, imaging is becoming the preferred method of diagnosis. MRI is timely and costly and not always readily available. A recent systematic review of 1168 studies and metanalysis including 10 studies reported that both TVUS and MRI have good diagnostic potential in diagnosis of adenomyosis [33]. A major strength of their analysis was the use of studies only with a proper comparison group: those with histopathology obtained following hysterectomy. Additionally, there was no statistically significant difference between the diagnostic ability of these imaging techniques. When TVUS was compared to MRI-confirmed adenomyosis, the reported overall diagnostic specificity was 91.8% and 99.1%, respectively, for women with 6+ sonographic findings [34]. Thus, positive findings are likely to be a true positive diagnosis. Recently, the more widespread use of 3D-TVUS offers the advantage of offline examination and manipulation of images. 3D-TVUS provides, in addition, a detailed display of the junctional zone, specifically by allowing for lateral and fundal assessment [8,35,36]. In support of our findings, the use of combined 2D/3D-TVUS has been shown to increase the accuracy and specificity of adenomyosis diagnosis [35,37]. Exacoustos et al. reported a higher incidence of infertility and miscarriage in adenomyosis of the JZ junction [38]. It should be noted, however, that the lack of a standardized reporting system for sonographic findings associated with adenomyosis translated to a large amount of heterogeneity in the previously reported data related to adenomyosis, making the comparison of studies difficult. Therefore, the publishing of the consensus statement in 2015 offered clinicians a much more homogenous approach to reporting and diagnosing adenomyosis [24], followed by a scoring system for the diagnosis of adenomyosis based on their Morphological Uterus Sonographic Assessment (MUSA)-defined criteria [32].

Bluhm et al. highlighted the benefit of this newly defined system and even published video guidance on the evaluation of the MUSA criteria [39]. The combined use of both 2D/3D-TVUS should be considered a viable noninvasive diagnostic tool for adenomyosis. Other groups have created scoring systems for reporting the MUSA criteria and have shown interobserver reproducibility [40]. It seems that a standardized method of reporting sonographic findings of adenomyosis should be evaluated in the future in prospective studies.

The strengths of the current study are that a single operator dedicated to comprehensive endometriosis and adenomyosis evaluation performed all of the TVUS examinations, using a high-frequency transvaginal probe and utilizing uniform validated diagnostic criteria for all patients, which were recently described in a statement paper by the Myometrial Pathology Using Ultrasonography Consensus Group [24]. The inclusion of a control group of women undergoing surgeries highlights an additional strength of our study.

The main limitations of this study were the retrospective design and the limited availability of histological confirmation of hysterectomy specimens, but as described above, ultrasound can offer valid noninvasive diagnosis, making surgical confirmation unnecessary. Most of those included in the study were young women seeking to preserve fertility, which explains the low hysterectomy rate. This would inevitably affect their motivation for undergoing surgery or not, and thus affect the study groups. This limitation would be difficult to control for other than the fact that the two groups did not differ significantly in their demographic parameters, with the exception of parity, which we discussed above. Another limitation is that the study population was composed of women with severe endometriosis, who were seen in a tertiary endometriosis referral center and thus were not representative of the entire population. Accordingly, the high prevalence of adenomyosis in these patients might reflect a selection bias. Despite this limitation, we feel that the findings are striking enough to merit consideration when devising patient-specific management.

## 5. Conclusions

In conclusion, this study showed that sonographic signs of adenomyosis are more prevalent in women undergoing surgery for endometriosis compared to those who continued conservative management. Moreover, a higher risk of infertility was associated with increasing sonographic signs of adenomyosis, regardless of the severity of endometriosis. Therefore, we suggest that endometriosis severity is not the only predictor of infertility in these women, and that severe adenomyosis should be considered as a possible causative or prognostic factor. We suggest adding to the common classification a classification based on the number of adenomyosis findings, with more than five considered severe. Furthermore, the higher infertility rate among women who continued conservative management compared to those who underwent surgery, may suggest the need for earlier laparoscopic intervention, which is likely to alleviate pain as well as infertility. We believe that our findings may have direct implications on clinical practice when designing patient-specific fertility treatments, both before and after surgery, such as secondary prevention using hormonal therapy, or choice and timing of fertility treatments.

## Figures and Tables

**Table 1:** Demographic data, history, and symptoms of women who underwent TVUS and subsequent laparoscopic surgery for endometriosis compared with those who did not undergo surgery.

Characteristics	Surgery (*n* = 244)	No Surgery (*n* = 158)	*p*-Value
Age at ultrasound, mean ± SD, years	32.8 ± 6.5	32.4 ± 6.8	ns
BMI, mean ± SD, kg/m^2^	23.1 ± 4.6	25.1 ± 7.6	ns
Smoker pack years N (%)	31.8	29.1	ns
Parous N (%)	39.3	28.5	0.03
Parity, mean ± SD,	0.78 ± 1.14	0.61 ± 0.99	ns
Median (range)	0 (0–6)	0 (0–4)	
Previous cesarean section N (%)	14.3	12.7	ns
No. of laparoscopies, mean ± SD,	0.7 ± 1.1	0.5 ± 0.8	ns
Median and range	0 (0–12)	0 (0–3)	
Dysmenorrhea (%)	92.3	86.6	ns
Dyspareunia (%)	68.3	63.4	ns
Urinary symptoms (%)	31.6	30	ns
GI symptoms (%)	61.2	61	ns
Infertility (%)	57.5	74.1	ns
Previous IVF treatments (%)	37	55	ns
No. of IVF cycles, mean ± SD,	2.2 ± 4.1	3.2 ± 5.2	ns
Median and range	0 (0–20)	2 (0–20)	
CA125, mean ± SD, U/Ml	69.5 ± 66.6	37.6 ± 53.9	0.02

SD—Standard deviation; BMI—Body mass index; IVF—In vitro fertilization.

**Table 2:** TVUS findings suggestive of adenomyosis in women undergoing surgery for endometriosis versus no surgery.

Ultrasound Findings	Surgery (*n* = 244)	No Surgery (*n* = 158)	*p*-Value
Asymmetrical myometrial thickening (%)	148 (60.7)	70 (44)	0.001
Myometrial cysts (%)	160 (65.6)	74 (46.8)	<0.001
Parallel shadowing (%)	125 (51.2)	59 (37.1)	0.008
Hyperechoic islands (%)	169 (69.3)	71 (44.9)	<0.001
Linear striations (%)	63 (25.8)	31 (19.6)	ns
Irregular EMJ (%)	180 (73.8)	79 (0.5)	<0.001
Focal adenomyomas (%)	67 (27.5)	40 (25.3)	ns
Any one feature (%)	190 (77.9)	102 (64.6)	0.004
3+ findings (%)	179 (73.3)	80 (50.1)	<0.001
5+ findings (%)	112 (45.9)	44 (27.8)	<0.001
No. of findings, mean ± SD	3.73 ± 2.3	2.7 ± 2.4	<0.001

EMJ—Endometrial-myometrial junction; SD—Standard deviation.

**Table 3:** Odds ratios for the association between sonographic findings of adenomyosis in women undergoing surgery versus those who did not, and demographic variables using logistic regression for three chosen models: unadjusted-without adjustment for variables; and adjusted for age.

Sonographic Findings	Unadjusted				Adjusted for Age			
		95% CI for OR				95% CI for OR		
	OR	LL	UP	*p*-Value	OR	LL	UL	*p*-Value
Any one finding	1.93	1.24	3.01	0.004	1.07	1.03	1.11	<0.001
3+ findings	2.69	1.76	4.09	<0.001	1.05	1.02	1.09	0.002
5+ findings	2.20	1.43	3.38	<0.001	1.05	1.02	1.08	0.004
Asymmetrical thickening	1.94	1.29	2.91	0.001	1.05	1.02	1.08	0.003
Myometrial cysts	2.16	1.44	3.26	<0.001	1.04	1.01	1.08	0.008
Parallel shadowing	1.76	1.17	2.65	0.007	1.05	1.01	1.08	0.005
Hyperechoic islands	2.76	1.82	4.08	<0.001	1.05	1.02	1.09	0.001
Linear striations	1.43	0.88	2.33	Ns	1.04	1.00	1.07	0.045
Irregular EMJ	2.81	1.84	4.29	<0.001	1.05	1.02	1.09	0.02
Focal adenomyomas	1.12	0.71	1.76	Ns	1.05	1.01	1.08	0.01

CI—confidence interval; OR—odds ratio; LL—lower limit; UL—upper limit; EMJ—endometrial-myometrial junction.

**Table 4:** Univariate analysis of the associations between demographic data, clinical symptoms, and the number of sonographic findings of adenomyosis in women undergoing surgery (*n* = 244).

	3+ Findings				5+ Findings			
Variable		95% CI for OR				95% CI for OR		
	OR	LL	UL	P	OR	LL	UL	P
Age	1.04	0.99	1.09	0.08	1.03	0.99	1.07	ns
BMI	1.02	0.96	1.09	ns	1.01	0.96	1.07	ns
Smoker	1.07	0.58	1.98	ns	0.72	0.41	1.24	ns
Parity	1.83	0.99	3.37	0.05	0.81	0.48	1.36	ns
Previous cesarean section	0.76	0.35	1.65	ns	0.66	0.31	1.37	ns
No. of laparoscopies	0.90	0.70	1.16	ns	1.00	0.79	1.27	ns
Dysmenorrhea	0.83	0.26	2.63	ns	0.61	0.34	1.10	ns
Dyspareunia	0.65	0.32	1.32	ns	0.70	0.44	1.11	ns
Urinary symptoms	0.93	0.44	1.99	ns	0.65	0.34	1.24	ns
GI symptoms	0.35	0.36	1.43	ns	1.22	0.68	2.17	ns
Infertility	0.71	0.33	1.53	ns	2.31	1.20	4.45	0.012
Previous IVF treatments	0.66	0.29	1.51	ns	2.05	1.00	4.20	0.05
No. of IVF cycles	1.23	0.93	1.14	ns	1.14	1.03	1.26	0.014
CA125	0.99	0.99	1.00	ns	1.00	1.00	1.00	ns

BMI—body mass index; IVF—In vitro fertilization, GI—gastrointestinal; OR—odds ratio; LL—lower limit; UL—upper limit.

## Data Availability

Not applicable.
